# Comparison of Recruitment Strategies for Engaging Older Minority Adults: Results From Take Heart

**DOI:** 10.1093/geroni/igab046.1328

**Published:** 2021-12-17

**Authors:** Jessica Ramsay, Cainnear Hogan, Mary Janevic, Rebecca Courser, Kristi Allgood, Cathleen Connell

**Affiliations:** 1 University of Michigan School of Public Health, Detroit, Michigan, United States; 2 VA Ann Arbor Healthcare System, Ann Arbor, Michigan, United States; 3 University of Michigan School of Public Health, Ann Arbor, Michigan, United States

## Abstract

Few studies report best practices for recruiting older adults from minority, low SES communities for behavioral interventions. In this presentation, we describe recruitment processes and numbers for Take Heart, a randomized controlled trial testing the effectiveness of an adapted heart disease self-management program for primarily African American, low SES adults 50 years or older in Detroit. Community-based (CB), electronic medical record (EMR), and in-person hospital clinic (HC) recruitment methods were implemented. Within 22 months, 453 participants were enrolled, with an overall recruitment yield of 37%. The CB method had the highest yield (49%), followed by HC (36%) and EMR (16%). The average cost of recruiting and enrolling one participant was $142. Face-to-face interactions and employing a community health worker were particularly useful in engaging this population. Further research is needed to confirm these findings in other minority and low SES populations and share lessons learned about recruitment challenges and successes.

